# Psychometrics of the Greek Chronic Liver Disease Questionnaire for Measuring HRQL

**DOI:** 10.1155/2015/395951

**Published:** 2015-07-30

**Authors:** Evelina Pappa, Fotini Hatzi, Angelos A. Papadopoulos, Dimitris Niakas

**Affiliations:** ^1^Faculty of Social Sciences, Hellenic Open University, 18 Parodos Aristotelous, 26335 Patras, Greece; ^2^ATTIKON University Hospital, 1 Rimini, 12462 Athens, Greece

## Abstract

The aim of the present study is to examine psychometric properties such as internal consistency reliability and construct validity of the Greek CLDQ. A sample of 366 eligible patients with chronic liver disease (CLD) self-administered the Greek version of the SF-36 Health Survey, the Chronic Liver Disease Questionnaire (CLDQ), and questions on sociodemographic status and treatment. Child Pugh Score was also collected. Hypothesized scale structure, reliability (Cronbach's alpha), and construct validity (convergent, discriminant, and known groups) were assessed. Multitrait scaling confirmed scale structure of the CLDQ with good item convergence (100%) and discrimination (84.1%) rates. Cronbach's alpha rated >0.70 for all scales. Spearman's correlations between the CLDQ and SF-36 scales assessing similar health-related quality of life dimensions were strong ranging above 0.70 (*P* < 0.0001). Construct validity was confirmed with satisfactory results for known-group comparisons. Most CLDQ scales discriminated significantly between patients according to disease severity, whereas all CLDQ scales discriminated between treatment receivers and nonreceivers. The overall psychometric results for the Greek version of the CLDQ confirmed it as a reliable and valid questionnaire.

## 1. Introduction

Chronic liver disease (CLD) is a serious illness which not only causes high mortality and morbidity but also affects negatively the quality of life. Patients with CLD experience a variety of symptoms such as fatigue, anxiety, as a result of disease progression which have a profound negative impact on health-related quality of life (HRQL) [1, 2]. In last decades, there is an increasing interest in assessing the impact of chronic diseases on peoples' HRQL. The rising prevalence of chronic diseases in developed countries has led to an increased focus on the emotional, social, and physical well-being [3]. Emphasis is given to the importance of HRQL in assessing chronic disease's outcomes and the impact of interventions [4]. Generic and disease-specific instruments have been used for measuring the impact of chronic diseases on HRQL. Although generic questionnaires measure HRQL among different chronic diseases, they may not be responsive enough to detect small but clinically important changes due to disease progression or interventions and therefore they are complementary in assessing the total impact of a chronic disease on HRQL [4, 5].

Disease-specific instruments for CLD have been recently developed. Chronic Liver Disease Questionnaire (CLDQ) [6] is the most widely used disease-specific questionnaire concerning patients with different etiology of chronic liver disease. It has been translated and validated in many countries [7–12] and findings have shown that the questionnaire has high reliability and validity results as well as good acceptability from the patients.

In Greece, the assessment of HRQL in patients with chronic liver disease had been previously investigated in patients with hepatitis B and hepatitis C using only the generic instrument of SF-36 [13–15]. CLDQ has been recently translated in Greek language, determining the dimensional structure of the questionnaire via factor analysis and assessing its sensitivity [16]. However, information on the validity and other psychometric properties is limited. In this context, the aim of the present study is to examine psychometric properties such as internal consistency reliability and construct validity of the Greek CLDQ with a twofold added value to fill in the gap in liver disease-specific questionnaires of HRQL and to contribute to the existing international knowledge on the subject.

## 2. Materials and Methods

### 2.1. Study Design and Population

The study was carried out at the Hepatological Department of General Hospital of West Attica, Greece, during January to April 2012, and involved a sample of 366 eligible patients with chronic liver disease (CLD). Patients with malignancies, other chronic diseases such as kidney or health failure, and autoimmune or pharmaceutical liver disease that may affect the HRQL were excluded from the study. Patients with psychiatric problems and language or cognitive difficulties that prevented reliable completion of the questionnaires were also excluded. Patients enrolled in the study were asked to self-administer the Greek versions of the Short Form 36 Health Survey (SF-36), the Chronic Liver Disease Questionnaire (CLDQ) and further to answer questions concerning sociodemographic characteristics. Data regarding the etiology of the CLD, the occurrence and the duration of the treatment, and biochemistry testing results for the assessment of Child Pugh Score were collected from the medical records. All the patients agreed to participate in the study after full notification. The study was approved by the hospital's Ethical Committee.

### 2.2. Questionnaires

CLDQ is a disease-specific, self-administered questionnaire which has been developed to assess HRQL in patients with CLD and particularly to measure small but important changes in HRQL where generic instruments of HRQL may fail to detect them [6]. It contains 29 items divided into six domains: Abdominal Symptoms (AS), Fatigue (FA), Systemic Symptoms (SS), Activity (AC), Emotional Function (EF), and Worry (WO) and a total scale of CLDQ whereas it measures in a seven-point scale the best possible function with the higher scores reflecting better HRQL. CLDQ has been translated in Greek language [16] and results from previous studies have supported the psychometric properties of the questionnaire [8, 9, 11].

SF-36 is a standardized, self-administered generic questionnaire validated in Greek language previously [17, 18]. It is widely used in health services research to record functional health status and general health perceptions. It is comprised of eight scales: Physical Functioning (PF), Role Limitations due to Physical Problems (RP), Bodily Pain (BP), General Health (GH), Vitality (VT), Social Functioning (SF), Role Limitations due to Emotional Problems (RE), and Mental Health (MH) with higher scores (0–100 range) reflecting better-perceived health. The eight dimensions can be summarized in two summary scores of physical and mental health [19] and it was used in the present study as standard for assessing HRQL.

### 2.3. Statistical Analysis

Percentages of floor and ceiling scores were provided since an instrument's ability to detect changes over time is constrained by the percentage of ceiling and floor scores [20]. Scale internal consistency was assessed via Cronbach's alpha and the 0.70 standard for the group-level comparisons was adopted [21]. Item convergent validity (which is substantial when correlation between an item and its hypothesized scale, corrected for overlap, is 0.40) and item-discriminant validity (which is successful when correlation between an item and its own scale is significantly higher, >2 standard errors, than with other scales) [20] were used to examine the hypothesized scale structure.

Spearman's correlation between CLDQ and SF-36 scales was used to assess convergent construct validity. Based on the literature, scales measuring similar HRQL dimensions were hypothesized to be strongly correlated [9, 11, 22] and convergent validity was fulfilled when the scale score for related concepts such as FA (CLDQ) and VT (SF-36), AC (CLDQ) and PF (SF-36), and EM (CLDQ) and MH (SF-36) is ranged above 0.7. Tests of “known groups” validity were implemented to provide supporting information for assessing the ability of the questionnaire to distinguish between subgroups of respondents known to differ in key sociodemographic or clinical variables. In this study, known-group comparisons were used to evaluate how well scales could discriminate between patients according to the severity of disease based on Child Pugh Score (classification of patients as noncirrhotics, early cirrhotics, Child's A, and advanced cirrhotics, Child's B and Child's C) [23] and to treatment status (receivers versus nonreceivers). Independent samples *t*-tests and ANOVA examined the statistical significance of group comparisons in *P* level > 0.05. All statistical analyses were performed with SPSS v. 17.0.

## 3. Results

Sociodemographic and medical characteristics of the sample are shown in Table 1. The majority of the patients were men (65%) and the ages varied between 19 and 86 years with a mean age of 48.8 (14.0). The majority were married (51.4%), were employed (57.1%), and have completed the secondary education. Of the 366 patients with CLD, 201 (54.9%) had chronic hepatitis and the remainder had cirrhosis divided into three classes according to severity of the disease (Child Pugh Score). Viral hepatitis B (44.5%) was the most prevalent etiology followed by viral hepatitis C (40.2%), being alcoholic (8.7%), and primary biliary cirrhosis (6.6%), whereas 71.6% of the patients had received treatment.

Data on central tendency and reliability of CLDQ scales are presented in Table 2. The distribution of the Greek CLDQ showed very low floor scores, whereas the ceiling scores were low to moderate with exception in Abdominal Symptoms (AS) scale (35%) and Activity (AC) scale (28.1%). Reliability measured by Cronbach's alpha ranged from 0.74 in Activity (AC) scale to 0.92 in Fatigue (FA) scale exceeding, in all cases, the 0.7 internal consistence criterion.

Significantly higher item-scale correlations between items and their hypothesized scales than with competing scales were observed in Table 3. The 0.40 item-scale correlation criterion was satisfied, confirming 100% item convergence in 29/29 tests for CLDQ scales. Accordingly, item discrimination was successful in 122/145, with scaling success rate in 84.1%.

High correlations between pairs of CLDQ and SF-36 scales measuring similar dimensions were confirmed in Table 4. “Fatigue” with “Vitality” (0.846), “Emotional Function” with “Mental Health” (0.824), and “Activity” with “Physical Functioning” (0.744) presented the highest correlations. All hypothesized strong correlations were statistically significant (*P* < 0.01). Other two pairs between CLDQ and SF-36 scales, that is, “Emotional Function” with “Vitality” (0.798) and “Fatigue” with “Social Functioning” (0.799), showed strong correlation as well. Concerning the other correlations, those hypothesized as moderate were above 0.5, whereas those hypothesized as noncomparable were up to 0.50.

Significant differences were detected in the CLDQ score distribution according to severity of disease and treatment receipt (Table 5). Patients without cirrhosis reported higher scores in all CLDQ scales compared to cirrhotic patients divided into Child Pugh groups, except Emotional Functioning and Worry, where patients with Child Pugh A reported better HRQL. Concerning cirrhotic patients, statistically significant differences were obtained in all scales, as it was expected, with patients with Child Pugh A reporting better HRQL than patients with Child Pugh B or C. Additionally all CLDQ subscale scores were significantly higher in treatment-receiving group rather than in no treatment-receiving group.

## 4. Discussion

CLDQ is a disease-specific, self-administered questionnaire, which was developed to assess HRQL in patients with CLD and has been translated and validated in many countries differing in culture [7–11] with remarkable results. The CLDQ was also translated in Greek language [16] according to the documented procedure. However, it has not been used in studies in Greece so far and therefore much space is available to fill in the existing gap in validation as well as to contribute to the international knowledge on the subject.

Psychometric properties of the questionnaire have shown satisfactory results implying good discriminative ability and probable good responsiveness as well. The very low floor scores indicate that the Greek CLDQ would be able to capture any deterioration in patients' QOL as the disease progresses. High ceiling scores were observed in two scales AS and AC, a finding which is common with previous studies [9, 22]. Treatment or possible adjustment of patients to their illness could be an explanation [22]. Reliability of the CLDQ was confirmed via the internal consistency. The results have shown that Cronbach's alpha exceeded the 0.70 criterion in all CLDQ scales for the group-level comparisons, providing evidence that each scale is measuring a similar underlying construct.

Multitrait scaling confirmed the hypothesized scale structure, implying that the translation of the items and the response choices are appropriate and that scale scores derived from the Greek version could contribute to cross-cultural comparisons. Our findings revealed that CLDQ has excellent convergent and discriminant validity whereas the results are comparable with those of previous studies on CLDQ validation [9, 11].

Criterion-related construct validity was confirmed by correlations between CLDQ and SF-36 scales measuring similar dimensions. CLDQ scales were significantly correlated with SF-36 scales. The patterns of the relationships between scales measuring similar dimensions followed the expected high correlations for FA, AC, and EM, a finding which is common to previous studies [9, 11]. Moreover, in our study, the pairing of “Emotional Function” with “Vitality” and “Fatigue” with “Social Functioning” showed a strong correlation as well. A possible explanation for that could be that scale captions implied conceptual similarities. Items of EF such as “unhappy” or “mood swings” or “depressed” can be conceptually related with items of VT such as “full of pep” or “worn out.” Alike, items of FA scale such as “decreased strength,” “decreased level of energy,” or “felt sleepy during the day” can be considered as physical or emotional problems interfering with social activities (SF scale). Low correlations (less than 0.5) were found between WO and some of the SF-36 scales.

In the analysis of known-group comparisons all scales were proved to be able to distinguish between groups differing in disease status (without cirrhosis and cirrhosis, Child Pugh stage, and treatment status, receivers and nonreceivers). The results were in agreement with the hypothesis that remarkable decrease in QOL was recorded in advanced stages of chronic liver disease and in patients without receiving any treatment, corresponding also to previous findings [1]. A distinct and significant deterioration in QOL between the three stages is evident, enhancing the discrimination ability. On the other hand, it has been noticed that CLDQ EF and WO scales were significantly lower in patients with chronic hepatitis than those with Child Pugh A cirrhosis. A possible explanation could be that, in some parts, chronic hepatitis may impair HRQL more than Child Pugh A, which may be a more stable condition [7]. However, our results agree with findings from previous studies that deterioration of HRQL may not be in step with the severity of disease and that other factors may also contribute.

Using the CLDQ along with generic instruments not only will allow the identification of changes in HRQL, a highly important fact to patients, but also will facilitate comparisons across different disease stages contributing to cross-sectional studies as well. The administration of the study to a cohort of patients differing in types and stages of CLD and the derived deterioration of HRQL according to the severity of disease supports the construct validity of the CLDQ as a cross-sectional measure of HRQL for CLD [6]. One possible limitation could be that study participants were recruited from one referral outpatient center and hospitalized patients with severe diseases were not included.

## 5. Conclusions

CLDQ demonstrates adequate psychometric properties; our findings support the reliability and validity of the questionnaire that can be used in clinical practice assisting health professionals to measure functional status, well-being and hence further improve patients' HRQL.

## Figures and Tables

**Table 1:** Sociodemographic characteristics of the population study.

Variables	*N*	%
Sex		
Men	238	65.0
Women	138	35.0
Age		
19–24	9	2.5
25–34	61	16.7
35–44	68	18.6
45–54	95	26.0
55–64	73	19.9
65+	60	16.4
Marital status		
Single	178	48.6
Married	188	51.4
Education		
Primary	128	27.6
Secondary	150	41.0
University	88	24.0
Occupation		
Employers	102	27.9
Employees	107	29.2
Retired	40	10.9
Other	117	32.0
Severity of disease		
No cirrhosis	201	54.9
Child's Pugh A	88	24.0
Child's Pugh B	54	16.8
Child's Pugh C	23	6.3
Etiology of the liver disease		
Hepatitis B	163	44.5
Hepatitis C	147	40.2
Alcoholic	32	8.7
Primary biliary cirrhosis	24	6.6
Treatment		
Yes	262	71.6
No	104	28.4

**Table 2:** Central tendency, variability, and reliability of the CLDQ scales.

	*N*	Mean (SD)	95% CI	Median	Ceiling^§^	Floor^¶^	Reliability^*∗*^
AS	3	5.66 (0.72)	5.52–5.81	6.00	35.0	0.3	0.89
FA	5	5.36 (0.78)	5.21–5.51	5.60	24.9	0.3	0.94
SS	5	4.98 (0.61)	4.86–5.10	5.20	2.5	0.3	0.76
AC	3	5.79 (0.60)	5.67–5.91	6.00	28.1	0.3	0.74
EF	8	4.95 (0.76)	4.80–5.10	5.25	11.7	0.3	0.82
WO	5	5.54 (0.54)	5.44–5.65	5.60	11.7	0.3	0.92
CLDQ		5.38 (0.57)	5.27–5.50	5.61	0.3	0.3	0.92

^§^Highest scores.

^¶^Lowest scores.

^*∗*^Cronbach's alpha.

AS = Abnormal Symptoms, FA = Fatigue, SS = Systemic Symptoms, AC = Activity, EF = Emotional Function, and WO = Worry.

**Table 3:** Summary results of scaling assumptions tests.

	*N* ^*∗*^	Item-internal consistency		Item-discriminant validity	
		Range of correlations^†^	Success/total^‡^	Range of correlations^§^	Success/total^¶^
AS	3	0.77–0.87	3/3	0.46–0.75	15/15
FA	5	0.73–0.88	5/5	0.40–0.81	25/25
SS	5	0.41–0.65	5/5	0.27–0.86	16/25
AC	3	0.45–0.70	3/3	0.24–0.84	10/15
EF	8	0.76–0.85	8/8	0.39–0.86	36/40
WO	5	0.53–0.76	5/5	0.08–0.81	20/25

^*∗*^Number of items and number of internal consistency tests per scale.

^†^Range of correlations between item and hypothesized scale corrected for overlaps.

^‡^Number of correlations exceeding the 0.40 standard/total number of correlations.

^§^Range of correlations between items and other scales.

^¶^Number of successful discriminant validity tests/total number of discriminant validity tests.

AS = Abnormal Symptoms, FA = Fatigue, SS = Systemic Symptoms, AC = Activity, EF = Emotional Function, and WO = Worry.

**Table 4:** Spearman's rank correlations between CLDQ and SF-36 scales.

	AS	FA	SS	AC	EF	WO	CLDQ
PF	0.597	0.762	0.691	0.744	0.636	0.438	0.756
RP	0.643	0.717	0.646	0.667	0.614	0.480	0.735
BP	0.662	0.763	0.702	0.686	0.660	0.475	0.776
GH	0.595	0.614	0.563	0.546	0.586	0.538	0.676
VT	0.648	0.846	0.727	0.608	0.798	0.560	0.825
SF	0.667	0.799	0.759	0.697	0.726	0.536	0.825
RE	0.572	0.702	0.589	0.568	0.666	0.496	0.708
MH	0.598	0.778	0.655	0.524	0.824	0.506	0.771

AS = Abnormal Symptoms, FA = Fatigue, SS = Systemic Symptoms, AC = Activity, EF = Emotional Function, and WO = Worry.

PF = Physical Functioning, RP = Role Physical, BP = Bodily Pain, GH = General Health, VT = Vitality, SF = Social Functioning, RE = Role Emotional, and MH = Mental Health.

**Table 5:** Comparisons of CLDQ scores according to the severity of liver disease and receipt of treatment.

	AS	FA	SS	AC	EF	WO
Severity of disease						
No cirrhosis	5.90	5.62	5.21	6.13	5.09	5.54
Child's Pugh A	5.71	5.48	4.95	5.65	5.13	5.76
Child's Pugh B	5.14	4.79	4.65	5.22	4.59	5.41
Child's Pugh C	4.65	3.88	3.80	4.62	3.89	4.97
Sig	*0.0001 *	*0.0001 *	*0.0001 *	*0.0001 *	*0.0001 *	*0.007 *
Treatment						
Yes	5.79	5.57	5.16	5.87	5.16	5.65
No	5.33	4.83	4.53	5.59	4.43	5.27
Sig	*0.004 *	*0.0001 *	*0.0001 *	*0.037 *	*0.0001 *	*0.001 *

AS = Abnormal Symptoms, FA = Fatigue, SS = Systemic Symptoms, AC = Activity, EF = Emotional Function, and WO = Worry.
